# Efficient minimization of multipole electrostatic potentials in torsion space

**DOI:** 10.1371/journal.pone.0195578

**Published:** 2018-04-11

**Authors:** Nicholas K. Bodmer, James J. Havranek

**Affiliations:** Department of Biochemistry and Molecular Biophysics, Washington University School of Medicine, St. Louis, Missouri, United States of America; Wake Forest University, UNITED STATES

## Abstract

The development of models of macromolecular electrostatics capable of delivering improved fidelity to quantum mechanical calculations is an active field of research in computational chemistry. Most molecular force field development takes place in the context of models with full Cartesian coordinate degrees of freedom. Nevertheless, a number of macromolecular modeling programs use a reduced set of conformational variables limited to rotatable bonds. Efficient algorithms for minimizing the energies of macromolecular systems with torsional degrees of freedom have been developed with the assumption that all atom-atom interaction potentials are isotropic. We describe novel modifications to address the anisotropy of higher order multipole terms while retaining the efficiency of these approaches. In addition, we present a treatment for obtaining derivatives of atom-centered tensors with respect to torsional degrees of freedom. We apply these results to enable minimization of the Amoeba multipole electrostatics potential in a system with torsional degrees of freedom, and validate the correctness of the gradients by comparison to finite difference approximations. In the interest of enabling a complete model of electrostatics with implicit treatment of solvent-mediated effects, we also derive expressions for the derivative of solvent accessible surface area with respect to torsional degrees of freedom.

## Introduction

Two aspects of molecular modeling under continual improvement are the accurate scoring and sampling of molecular conformations. Models for macromolecular electrostatics in particular have become increasingly more sophisticated over time. Multipole descriptions of electrostatics can provide representations of charge distributions that are orders of magnitude more faithful to distributions obtained from quantum mechanical calculations that those provided by atom-centered partial charges alone [1], although introducing off-atom partial charges also provides improvements in this respect [2,3]. Dipole moments also provide an attractive mechanism for introducing polarization of charge, which allows for a contextual dependence of electrostatics that is not possible when fixed partial charges are assigned to atoms irrespective of their exposure to polar solvents or their burial in hydrophobic environments. Modeling induced polarization is computationally taxing, but is highly relevant to molecular conformation [4] and is increasingly considered in forcefield development [5–7]. In addition, the electrostatic component of hydrogen bonding can be modeled by higher order moments, as the geometry of hydrogen bonds involving sp^2^-hybridized acceptors is poorly reproduced by atom-centered partial charges alone [8]. Recent computational and methodological advances have made such calculations viable for systems containing larger biomolecules including peptides [9,10], proteins [7,11–13], membranes [14], and nucleic acids [15–19].

**Fig 1:**
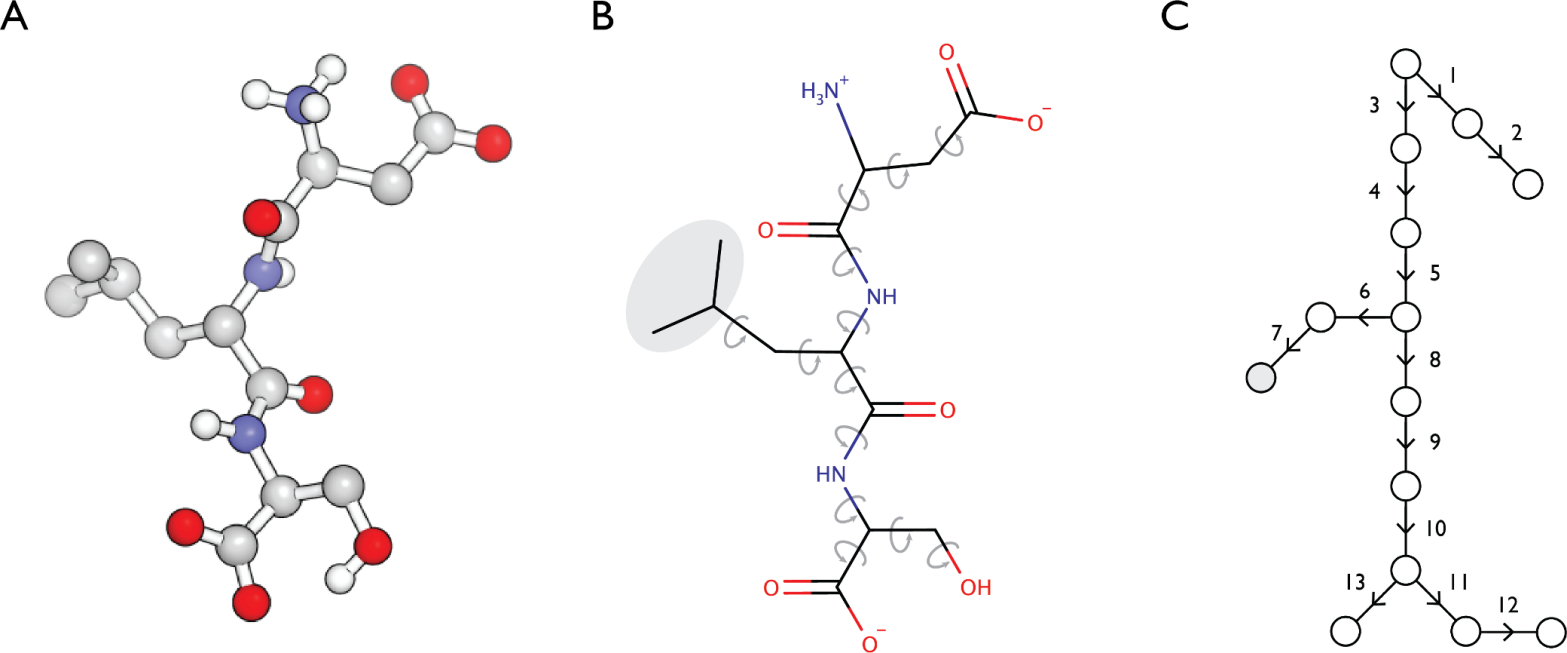
Tree-based representation of torsional degrees of freedom in a peptide. A. A tripeptide (sequence Asp-Leu-Ser) is shown in ball and stick representation. B. The same tripeptide is shown schematically. When bond lengths and angles are held constant, the conformation of the macromolecule is determined by the torsion angles at rotatable bonds, indicated by the circular grey arrows. Atoms that move as a coordinated rigid body may be grouped together, for example the three terminal carbon atoms in the leucine side chain (indicated by the gray oval). C. In a tree-based representation, atoms that necessarily rotate together are logically combined into single units denoted as circles. The gray circle downstream of torsion bond 7 corresponds to the unit consisting of the three atoms in the gray oval of panel B.

One strategy for enhancing sampling in molecular modeling is to utilize a reduced set of degrees of freedom in describing molecular conformation. A common selection for these degrees of freedom is the torsion angles of the rotatable bonds in the system (Fig 1A and 1B). This can reduce the number of degrees of freedom in a system by an order of magnitude, resulting in faster optimization of macromolecular geometry. In the case of molecular dynamics, elimination of higher frequency degrees of freedom such as bond vibrations allows for the use of time integration steps up to 10 times longer. Additionally, the degrees of freedom that are conserved in these models are often the most important when sampling interesting protein motions such as conformational change, thus increased efficiency does not come at the cost of reduced relevance [20–23]. Examples of molecular modeling programs that use a torsional representation include the NIH version of XPLOR [24], the CYANA program for NMR structure determination [25], the Rosetta molecular modeling program [26], and the ICM molecular modeling program, which uses an internal coordinate (torsions, angles, and bonds) description of macromolecular structure [27].

Multipole models for electrostatics have been implemented in systems that allow for full coordinate freedom for macromolecular atoms. For this combination of energy potential and conformational freedom, the Cartesian tensor formulation of multipole interactions is straightforward and efficient [28]. Of course, the same features of accurate, polarizable multipole electrostatics that make them attractive for full coordinate molecular mechanics apply to torsional systems as well. Efficient minimization of large systems with torsional degrees of freedom is enabled by the expression of the energy potential gradients in a recursive form that exploits the tree-based topology of molecular conformation [29] (Fig 1C). Gradients of atom-atom interactions must be expressed in a special form to be utilized within this framework, and the recursive procedure for summing atom-atom contributions to torsional gradients requires that all energy potentials be isotropic.

The limitation that only isotropic potentials are compatible with the logic of the tree-based minimization scheme must be addressed if we are to incorporate multipole electrostatics within this efficient framework. In addition, derivatives of atom-centered tensor quantities such as quadrupole moments with respect to arbitrary torsional degrees of freedom must be derived in a suitable form to be readily combined with the framework.

In this paper we address both of these concerns. First, we enable the inclusion of anisotropic potentials such as those of electrostatic dipole and quadrupole moments into the standard framework for torsional degrees of freedom by introducing the notion of orientational ‘reference atoms’. We also present the torsion-space derivatives required for electrostatic treatments including multipole terms out to quadrupoles. We have truncated our analysis at quadrupoles as we are unaware of any macromolecular force fields that make use of higher moments. We have selected for implementation the Amoeba polarizable, atomic multipole based electrostatics model because of its excellent agreement with quantum mechanical calculations and because its functional form is representative of similar models [7]. This model can be readily extended to handle solvent-mediated effects on electrostatics via the generalized Kirkwood model [30]. Implicit incorporation of the effects of solvent upon modeling calculations typically includes both solvent-mediated electrostatics and a surface area scaled term to describe the energetics of cavity formation. Therefore, in the interest of completeness we also present derivatives for the solvent accessible surface area of a macromolecule with respect to torsional degrees of freedom. We have implemented these potentials with analytical derivatives in the Rosetta molecular modeling program and verified their correctness by comparison to finite difference approximations with respect to torsion bond rotation.

## Materials and methods

### Vector formulation of multipole electrostatics

In macromolecular systems with full Cartesian degrees of freedom, multipole electrostatics can be put into a compact tensor formulation [28]. For systems with torsional degrees of freedom, however, it is more convenient to deal with expressions for the energetic interactions between atoms written explicitly in terms of the multipole moments themselves. These expressions can be obtained from multipole expansions of the electrostatic potential and its gradients, truncated at the desired moment. This functional form facilitates the calculation of derivatives with respect to rotatable bonds, which are more clearly written in terms of cross products of position vectors for mobile atoms with the bond’s axis of rotation. This results in a set of equations in vector form [28]. The monopole-monopole interaction between two atoms 1 and 2 is given by:

$$E_{ES}^{q-q}=\frac{q_{1}q_{2}}{r_{12}}$$ (1)

where the charge of each atom is denoted by *q*, and the distance between the atoms is given by *r*_12_. Inclusion of atomic dipoles leads to the following interactions between monopoles and dipoles:

$$E_{ES}^{q-\mu }=-\frac{q_{1}(\mu_{2}∙r_{12})}{r_{12}^{3}}+\frac{q_{2}(\mu_{1}∙r_{12})}{r_{12}^{3}}$$ (2)

where each atomic dipole moment is given by ***μ*** and the displacement vector between atoms 1 and 2 is given by ***r***_**12**_, as well as the following dipole-dipole interactions:

$$E_{ES}^{\mu -\mu }=\frac{\left(\mu_{1}∙\mu_{2}\right)}{r_{12}^{3}}-\frac{3(\mu_{1}∙r_{12})(\mu_{2}∙r_{12})}{r_{12}^{5}}.$$ (3)

Finally, atomic quadrupoles interact with monopoles, dipoles, and other quadrupoles:

$$E_{ES}^{q-\Theta }=\frac{q_{1}(r_{12}∙\Theta_{2}∙r_{12})}{r_{12}^{5}}+\frac{q_{2}(r_{12}∙\Theta_{1}∙r_{12})}{r_{12}^{5}}$$ (4)

$$E_{ES}^{\mu -\Theta }=-\frac{2(r_{12}∙\Theta_{2}∙\mu_{1})}{r_{12}^{5}}+\frac{2(r_{12}∙\Theta_{1}∙\mu_{2})}{r_{12}^{5}}+\frac{5(\mu_{1}∙r_{12})(r_{12}∙\Theta_{2}∙r_{12})}{r_{12}^{7}}-\frac{5(\mu_{2}∙r_{12})(r_{12}∙\Theta_{1}∙r_{12})}{r_{12}^{7}}$$ (5)

$$E_{ES}^{\Theta -\Theta }=\frac{20(r_{12}∙\Theta_{1}\Theta_{2}∙r_{12})}{{3r}_{12}^{7}}+\frac{35(r_{12}∙\Theta_{1}∙r_{12})(r_{12}∙\Theta_{2}∙r_{12})}{{3r}_{12}^{9}}+\frac{2\sum_{i,j}{\Theta_{1}^{i,j}\Theta }_{2}^{i,j}}{3r_{12}^{5}}.$$ (6)

In these equations the quadrupole moment is denoted by **Θ**, and the elements of the matrix representation of the quadrupole tensor are indexed with superscripts.

### Torsional minimization framework of Gō and coworkers

Calculation of the gradients of energy terms for a system with torsional degrees of freedom is complicated by the fact that the energy terms are typically defined in terms of atomic positions that are not collocated with the degrees of freedom themselves. This is in contrast to Cartesian systems, where the gradient is required at each atomic position, and can usually be determined by summing the interactions of the atom with all other atoms of the system. Noguti and Gō demonstrated that the contribution to the energy gradient at a torsion bond due to a pair of atoms can be expressed in a form that is independent of the identity of the torsion bond, eliminating the need to recalculate atom-atom interactions for each degree of freedom [31]. In later work, Gō and coworkers leveraged the fact that the interactions between pairs of atoms only contribute to the gradients of rotatable bonds that separate the atoms. When combined with a tree-based description of a macromolecular conformation (Fig 1C), this allows for the total gradient at a given torsion bond to be written recursively in terms of the gradients of downstream torsion bonds, allowing for the calculation of all gradients in a single pass through the tree topology with storage requirements that scale linearly with macromolecular size [29].

To leverage this framework for efficient calculation of energy gradients, the contribution of the interaction between atoms *i* and *j* to the energy derivative at torsion bond α must be written in the following form:

$$\frac{{\partial E}_{i,j}}{\partial \alpha }=f_{i,j}∙n_{\alpha }+g_{i,j}∙(n_{\alpha }\times r_{\alpha }).$$ (7)

Here, ***r***_*α*_ and ***n***_*α*_ denote respectively the position and unit direction of torsion bond α, and are independent of the atom pair. The same vectors (***f***_*i*,*j*_ and ***g***_*i*,*j*_) can also be used to construct derivatives with respect to bond angles and bond lengths [27]. This allows for the minimization of multi-molecule complexes *via* the introduction of a ‘virtual bond’ to describe the rigid-body orientation between individual molecules [32]. In the following, we derive the values of ***f***_*i*,*j*_ and ***g***_*i*,*j*_ for interactions between electrostatic multipoles through the quadrupole-quadrupole terms of the multipole expansion.

### Assembly of protein test set

We assembled a test set of 107 pdb files culled from Dunbrack’s Pisces server [33,34]. Our selection criteria consisted of structures with a minimum1.5 Å resolution and R-factors <0.25. All structures contained a maximum of 200 residues with sizes ranging from 50–200. We used one chain from each pdb file, removing additional chains in cases of complexes or structures with more than one chain in the asymmetric unit. Unless otherwise specified, we used our entire set of culled structures for our tests of energy and gradient calculations.

### Numerical approximation of energy gradient

For diagnostic purposes, the Rosetta program allows for the optional calculation of a finite-difference approximation to the energy gradient for each degree of freedom during minimization. This is accomplished by a simple ‘brute force’ approach. To obtain the finite-difference approximation to the gradient for a rotatable bond, the total energy for the macromolecule is evaluated for conformations resulting from rotations +/- a small perturbation about the bond from the conformation for which the analytical gradient is calculated. The difference in energies for these two perturbed conformations is divided by the angular difference at the perturbed degree of freedom to yield an approximation to the gradient. We report the results for the smallest default perturbation used for this procedure (+/- 0.001 degrees), which is expected to give the most accurate approximation to the actual gradient. In Table A in S1 Appendix we present results from larger perturbations that demonstrate the sensitivity of the numerical approximation to perturbation size.

A similar procedure was used to compare the analytically calculated gradients for multipole electrostatic terms to a numerical approximation at a more fine-grained level. For each rotatable degree of freedom, two conformations were constructed with the torsion bond rotated by +/- 0.001 degrees. All atom-atom interactions were calculated independently for the two conformations, and the change in the energy of interaction was divided by 0.002 degrees to obtain a finite difference approximation to the gradient at the rotatable bond due only to the atom-atom pair. The value for each atom-atom pair is compared to the analytic gradient that would be calculated at that bond if all other atoms in the macromolecule had values of zero for all multipole components. In cases where both atoms are on the same side of the degree of freedom, the value of the gradient is zero, and only combinations of atom pairs with degrees of freedom with non-zero gradient contributed to the analysis of deviations between analytical and numerical gradients.

We used a similar approach to examine the solvent accessible surface area gradients at a fine level. Because the surface area of a sphere can be occluded by more then one neighboring sphere, we cannot obtain atom-atom contributions to the gradient at each torsional degree of freedom. Instead, we calculate the independent contribution of the change in SASA due to a single atom upon the gradient at each degree of freedom. As above, we construct for each torsion bond two conformations by rotating the bond by +/- 0.001 degrees. The SASA for each atom is computed for the two conformations, and the change between the two is used to obtain a finite difference approximation to the gradient at the rotatable bond attributable to the single atom. This value is compared to the analytic gradient calculated considering only that atom and torsional degree of freedom.

## Results

### Derivatives of multipole electrostatic potential terms

Many of the derivatives necessary for computing the gradients of multipole electrostatics through quadrupole terms have been given in previous work [35], which we summarize here. For a given torsion axis located at ***r***_*α*_, with direction given by the normal vector ***n***_*α*_, the derivative of an arbitrary normal vector ***u*** with respect to rotation about the axis is:

$$\frac{\partial u}{\partial \alpha }=n_{\alpha }\times u.$$ (8)

For two atoms that are moved relative to one another upon rotation about the torsion axis, the derivative of the displacement vector ***r***_12_ = ***r***_2_ − ***r***_1_ is given by:

$$\frac{\partial r_{12}}{\partial \alpha }=-(n_{\alpha }\times r_{2}-n_{\alpha }\times r_{\alpha })$$ (9)

where ***r***_*α*2_ is the displacement from the torsion axis to the second atom. The derivative of the distance *r*_12_ between two atoms can be written as:

$$\frac{\partial r_{12}}{\partial \alpha }=\frac{\partial \sqrt{r_{12}∙r_{12}}}{\partial \alpha }=\frac{1}{r_{12}}[\left(r_{2}\times r_{1})∙n_{\alpha }+(r_{2}-r_{1})∙(n_{\alpha }\times r_{\alpha }\right)]$$ (10)

The derivatives for changes to dipole moments that result from torsion bond rotations are handled by Eq (8), as the dipole moments behave as vectors. However, we lack an analogous equation for the derivative of a quadrupole tensor with respect to rotation about an arbitrary axis. Derivatives for vector-tensor terms in which the tensors are constant have been described previously [35]. To deal with derivatives for non-constant tensors, we note that all multipole terms involving quadrupoles ultimately result in scalar quantities, with the quadrupole tensor forming inner products with vectors from both sides. We first introduce auxiliary vectors to represent the inner product of the tensor with each of the flanking vectors. Then we apply Eq (8) to obtain the derivative for each of the auxiliary vectors, expanding through application of the product rule. Concretely, for a term in the form (***a*** ∙ ***θ*** ∙ ***b***), with quadrupole moment ***θ***, and vectors ***a*** and ***b***, we obtain:

$$\frac{\partial (a∙\theta ∙b)}{\partial \alpha }=a∙\frac{\partial d}{\partial \alpha }+b∙\frac{\partial c}{\partial \alpha }=a∙(n_{\alpha }\times d)+b∙(n_{\alpha }\times c),$$ (11)

where ***c*** ≡ ***θ*** ∙ ***a*** and ***d*** ≡ ***θ*** ∙ ***b***.

The final quadrupole-quadrupole term in the multipole expansion for the electrostatic interaction between two atoms in Eq (6) is:

$$\frac{2\sum_{i,j}{\Theta_{1}^{i,j}\Theta }_{2}^{i,j}}{3r_{12}^{5}}.$$ (12)

This sum over element-wise products of two quadrupole tensors can be written in vector form as:

$$\frac{2}{3r_{12}^{5}}(x∙\Theta_{1}∙\Theta_{2}∙x+y∙\Theta_{1}∙\Theta_{2}∙y+z∙\Theta_{1}∙\Theta_{2}∙z),$$ (13)

where the quadrupole tensor elements and the orthonormal basis axes are all taken to be in the global frame. As above, calculation of the derivatives for this term can be accomplished through the use of auxiliary vectors.

Using the approach outlined in this section, a complete set of gradients for multipole electrostatics through the quadrupole terms has been derived and is given in Table 1. An example derivation of one of the dipole-quadrupole interaction terms that demonstrates the approach is given in S1 Appendix.

**Table 1:** Multipole energy term derivatives for Gō framework in vector form.

Term	Vector Form		Derivative
Monopole-monopole	$\frac{q_{1}q_{2}}{r_{12}}$	**f**	$\frac{-q_{1}q_{2}}{r_{12}^{3}}(r_{2}\times r_{1})$
		**g**	$\frac{-q_{1}q_{2}}{r_{12}^{3}}r_{12}$
Monopole-dipole	$-\frac{q_{1}(\mu_{2}∙r_{12})}{r_{12}^{3}}$	**f**	$\frac{-q_{1}}{r_{12}^{3}}(\mu_{2}\times {r_{1}}_{1})+\frac{3q_{1}(\mu_{2}∙r_{12})}{r_{12}^{5}}(r_{2}\times r_{1})$
		**g**	$\frac{-q_{1}{\mu_{2}}_{2}}{r_{12}^{3}}+\frac{3q_{1}(\mu_{2}∙r_{12})}{r_{12}^{5}}r_{12}$
Dipole-monopole	$\frac{q_{2}(\mu_{1}∙r_{12})}{r_{12}^{3}}$	**f**	$\frac{q_{2}}{r_{12}^{3}}(\mu_{1}\times r_{2})-\frac{3q_{2}(\mu_{1}∙r_{12})}{r_{12}^{5}}(r_{2}\times r_{1})$
		**g**	$\frac{q_{2}\mu_{1}}{r_{12}^{3}}-\frac{3q_{2}(\mu_{1}∙r_{12})}{r_{12}^{5}}r_{12}$
Dipole-dipole	$\frac{\left(\mu_{1}∙\mu_{2}\right)}{r_{12}^{3}}$	**f**	$\frac{\left(\mu_{1}\times \mu_{2}\right)}{r_{12}^{3}}+\frac{3(\mu_{1}∙\mu_{2})}{r_{12}^{5}}(r_{2}\times r_{1})$
		**g**	$-\frac{3(\mu_{1}∙\mu_{2})}{r_{12}^{5}}r_{12}$
Dipole-dipole	$-\frac{3(\mu_{1}∙r_{12})(\mu_{2}∙r_{12})}{r_{12}^{5}}$	**f**	$-\frac{3}{r_{12}^{5}}(\left(\mu_{1}∙r_{12})(\mu_{2}\times r_{1})+(\mu_{2}∙r_{12})(\mu_{1}\times r_{2}\right))+\frac{15}{r_{12}^{7}}(\mu_{1}∙r_{12})(\mu_{2}∙r_{12})(r_{2}\times r_{1})$
		**g**	$-\frac{3}{r_{12}^{5}}((\mu_{1}∙r_{12})\mu_{2}-(\mu_{2}∙r_{12})\mu_{1})+\frac{15}{r_{12}^{7}}(\mu_{1}∙r_{12})(\mu_{2}∙r_{12})r_{12}$
Monopole-quadrupole	$\frac{q_{1}(r_{12}∙\Theta_{2}∙r_{12})}{r_{12}^{5}}$	**f**	$\frac{2q_{1}}{r_{12}^{5}}(I_{2}\times r_{1})-\frac{5q_{1}(r_{12}∙\Theta_{2}∙r_{12})}{r_{12}^{7}}(r_{2}\times r_{1})$
		**g**	$\frac{2q_{1}}{r_{12}^{5}}I_{2}-\frac{5q_{1}(r_{12}∙\Theta_{2}∙r_{12})}{r_{12}^{7}}r_{12}$
Quadrupole-monopole	$\frac{q_{2}(r_{12}∙\Theta_{1}∙r_{12})}{r_{12}^{5}}$	**f**	$\frac{2q_{2}}{r_{12}^{5}}(I_{2}\times r_{2})-\frac{5q_{2}(r_{12}∙\Theta_{1}∙r_{12})}{r_{12}^{7}}(r_{2}\times r_{1})$
		**g**	$\frac{2q_{2}}{r_{12}^{5}}I_{2}-\frac{5q_{2}(r_{12}∙\Theta_{1}∙r_{12})}{r_{12}^{7}}r_{12}$
Dipole-quadrupole	$-\frac{2(r_{12}∙\Theta_{2}∙\mu_{1})}{r_{12}^{5}}$	**f**	$\frac{2}{r_{12}^{5}}(I_{2}\times \mu_{1})+\frac{10(I_{2}∙\mu_{1})}{r_{12}^{7}}(r_{2}\times r_{1})-\frac{2}{r_{12}^{5}}(J_{2}\times r_{1})$
		**g**	$-\frac{2}{r_{12}^{5}}J_{2}+\frac{10(I_{2}∙\mu_{1})}{r_{12}^{7}}r_{12}$
Quadrupole-dipole	$\frac{2(r_{12}∙\Theta_{1}∙\mu_{2})}{r_{12}^{5}}$	**f**	$\frac{2}{r_{12}^{5}}(I_{1}\times \mu_{2})-\frac{10(I_{1}∙\mu_{2})}{r_{12}^{7}}(r_{2}\times r_{1})+\frac{2}{r_{12}^{5}}(J_{1}\times r_{2})$
		**g**	$\frac{2}{r_{12}^{5}}J_{1}-\frac{10(I_{1}∙\mu_{2})}{r_{12}^{7}}r_{12}$
Dipole-quadrupole	$\frac{5(\mu_{1}∙r_{12})(r_{12}∙\Theta_{2}∙r_{12})}{r_{12}^{7}}$	**f**	$\frac{5(r_{12}∙\Theta_{2}∙r_{12})}{r_{12}^{7}}(\mu_{1}\times r_{2})+\frac{10(\mu_{1}∙r_{12})}{r_{12}^{7}}(I_{2}\times r_{1})-\frac{35(\mu_{1}∙r_{12})(r_{12}∙\Theta_{2}∙r_{12})}{r_{12}^{9}}(r_{2}\times r_{1})$
		**g**	$\frac{5(r_{12}∙\Theta_{2}∙r_{12})}{r_{12}^{7}}\mu_{1}+\frac{10(\mu_{1}∙r_{12})}{r_{12}^{7}}I_{2}-\frac{35(\mu_{1}∙r_{12})(r_{12}∙\Theta_{2}∙r_{12})}{r_{12}^{9}}r_{12}$
Quadrupole-dipole	$-\frac{5(\mu_{2}∙r_{12})(r_{12}∙\Theta_{1}∙r_{12})}{r_{12}^{7}}$	**f**	$\frac{-5(r_{12}∙\Theta_{1}∙r_{12})}{r_{12}^{7}}(\mu_{2}\times r_{1})-\frac{10(\mu_{2}∙r_{12})}{r_{12}^{7}}{\left(I_{1}\times r_{2})+\frac{35(\mu_{2}∙r_{12})(r_{12}∙\Theta_{1}∙r_{12})}{r_{12}^{9}}(r_{2}\times r_{1}\right)}_{12}$
		**g**	$\frac{-5(r_{12}∙\Theta_{1}∙r_{12})}{r_{12}^{7}}\mu_{2}-I_{1}+\frac{35(\mu_{2}∙r_{12})(r_{12}∙\Theta_{1}∙r_{12})}{r_{12}^{9}}r_{12}$
Quadrupole-quadrupole	$\frac{20(r_{12}∙\Theta_{1}\Theta_{2}∙r_{12})}{3r_{12}^{7}}$	**f**	$-\frac{20}{{3r}_{12}^{7}}(K_{1}\times r_{2}+K_{2}\times r_{1})+\frac{140(I_{1}∙I_{2})}{3r_{12}^{9}}(r_{2}\times r_{1})-\frac{20(I_{1}\times I_{2})}{3r_{12}^{7}}$
		**g**	$-\frac{20}{{3r}_{12}^{7}}(K_{1}+K_{2})+\frac{140(I_{1}∙I_{2})}{{3r}_{12}^{9}}r_{12}$
Quadrupole-quadrupole	$\frac{35(r_{12}∙\Theta_{1}∙r_{12})(r_{12}∙\Theta_{2}∙r_{12})}{3r_{12}^{9}}$	**f**	$\frac{70}{3r_{12}^{9}}({(r_{12}∙\Theta_{1}∙r_{12})I}_{2}\times r_{1}+(r_{12}∙\Theta_{2}∙r_{12})I_{1}\times r_{2})-\frac{105(r_{12}∙\Theta_{1}∙r_{12})(r_{12}∙\Theta_{2}∙r_{12})}{r_{12}^{11}}(r_{2}\times r_{1})$
		**g**	$\frac{70}{{3r}_{12}^{9}}({(r_{12}∙\Theta_{1}∙r_{12})I}_{2}+(r_{12}∙\Theta_{2}∙r_{12})I_{1})-\frac{105(r_{12}∙\Theta_{1}∙r_{12})(r_{12}∙\Theta_{2}∙r_{12})}{r_{12}^{11}}r_{12}$
Quadrupole-quadrupole	$\frac{2\sum_{i,j}{\Theta_{1}^{i,j}\Theta }_{2}^{i,j}}{3r_{12}^{5}}$	**f**	${-\frac{10T}{3r_{12}^{7}}{\left({r_{2}}_{2}\times r_{1}\right)}_{12}-\frac{4}{3r_{12}^{5}}([\Theta_{2}∙\Theta_{1}∙\hat{x}]\times \hat{x}+[\Theta_{2}∙\Theta_{1}∙\hat{y}]\times \hat{y}+[\Theta_{2}∙\Theta_{1}∙\hat{z}]\times \hat{z})}_{12}$
		**g**	$-\frac{10T}{3r_{12}^{7}}r_{12}$

The following definitions for auxiliary vectors are used in Table 1:

***I***_**(1/2)**_ = **Θ**_**(1/2)**_ ∙ ***r***_**12**_

***J***_**(1/2)**_ = **Θ**_**(1/2)**_ ∙ ***μ***_**2**_

***K***_**1**_ = **Θ**_**1**_ ∙ **Θ**_**2**_ ∙ ***r***_**12**_

***K***_**2**_ = **Θ**_**2**_ ∙ **Θ**_**1**_ ∙ ***r***_**12**_

$T=\sum_{i,j}\Theta_{1}^{i,j}\Theta_{2}^{i,j}$

### Frame rotation effects

**Fig 2:**
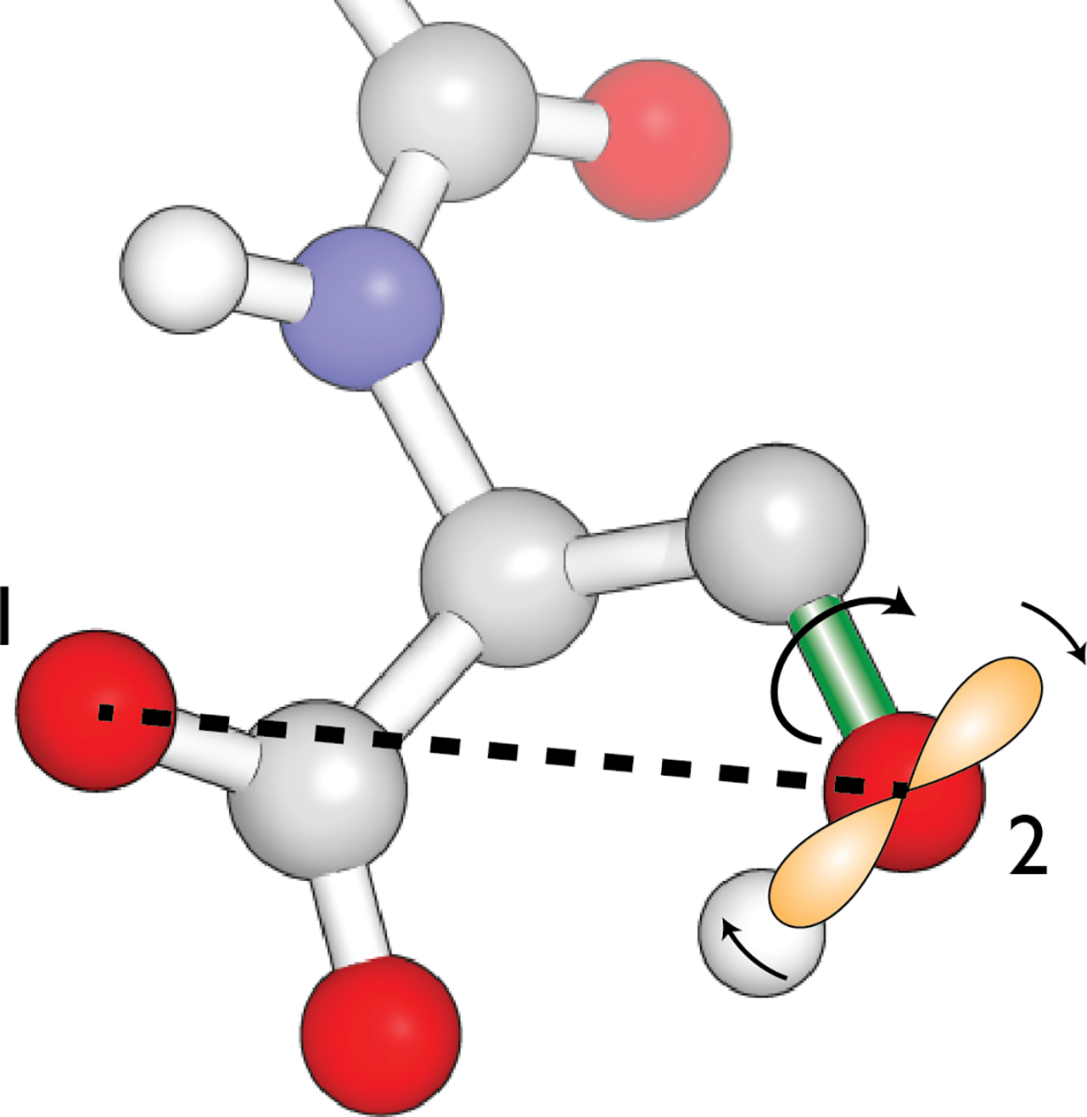
The frame rotation problem. Rotation about a torsion bond (for example, the bond colored green in the figure) changes the distances between atoms upstream and downstream of the bond. It also causes a rotation of the coordinate frame centered on the atom at the downstream end of the bond (atom 2 in the figure). This frame rotation results in a change in the dipole (denoted by the orange lobes) and quadrupole moments for the atom in the global frame. Thus, the energetic interaction between atoms 1 and 2 has a derivative with respect to the torsion angle even though the distance between the atoms remains constant.

In their original work, Gō and coworkers limited their attention to isotropic potentials that depend only on the distances between the atoms in a macromolecule. Under this assumption, rotation about a bond does not affect the energy of interaction between two atoms if both are either upstream or downstream of the atom that defines the end of the bond (Fig 2). This assumption is encoded in the groupings of atoms that are used for accumulating the energy gradient for each torsion angle in the recurrence relations presented by Gō and coworkers. However, in the case of electrostatics models that include dipole or higher moments, changes in orientation can have energetic consequences even for immobile atoms. While we need to include the energy gradient due to changes in orientation, it is also desirable to maintain the simplicity of the existing groupings of atoms and the logic governing interactions.

We have addressed this problem by introducing the notion of a ‘reference’ atom. A reference atom serves as a proxy for the local coordinate system of an atom: the orientation of a stationary atom rotates if and only if its reference atom rotates as well (e.g., the attached hydrogen for atom 2 in Fig 2). Using this notion, we incorporate orientational gradients into the existing framework as follows. When we calculate the gradient vectors **f** and **g** for the interaction between two atoms (1 and 2), we also calculate the portion of the derivative due only to rotation. This includes $\frac{\partial }{\partial \mu }$ and $\frac{\partial }{\partial \Theta }$, but neither $\frac{\partial }{\partial r}$ nor $\frac{\partial }{\partial r_{12}}$. This orientational gradient is now included in the overall gradient calculation twice. First, it is added to the gradient between atom 1 and the reference atom for atom 2. This will ensure that the orientational effect will be included even when atom 2 is fixed. However, this will also lead to double-counting of this part of the gradient when atom 2 moves with respect to atom 1. Therefore, we subtract the orientational gradient from the total gradient between atom 1 and atom 2. For torsions where both atom 2 and its reference atom are mobile with respect to atom 1, the two orientational modifications will cancel each other. However, for the torsion bond with atom 2 at its downstream end, the contributions will not cancel, and the correct contribution to the gradient due to the change in orientation of atom 2 will be incorporated *via* the modification applied to the (mobile) reference atom.

It should be noted that only atoms that are part of the folding path for the macromolecule (and thus serve as bounding atoms for a rotatable bond) require reference atoms. Because the Rosetta tree-based representation of conformation allows for folding in both directions, we identify reference atoms procedurally rather than specifying them as part of a residue definition. For each atom, we first look for attached hydrogen atoms to serve as the reference atom. If the atom is not bonded to any hydrogens, we next look for a bonded neighbor heavy atom that is not part of the folding pathway (e.g., the carbonyl oxygen of the peptide bond serves as the reference atom for the carbonyl carbon). This prescription is sufficient for the twenty canonical amino acids, yielding reference atoms that are appropriate regardless of the direction of folding along the polymer. In DNA and RNA, however, neither the O5’ nor O3’ atoms are bonded to suitable reference atoms. For these atoms we select the next downstream atom in the folding pathway as the reference atom; these selections must be updated whenever the direction of folding along the tree of atoms is changed.

### Split coordinate frame effect

**Fig 3:**
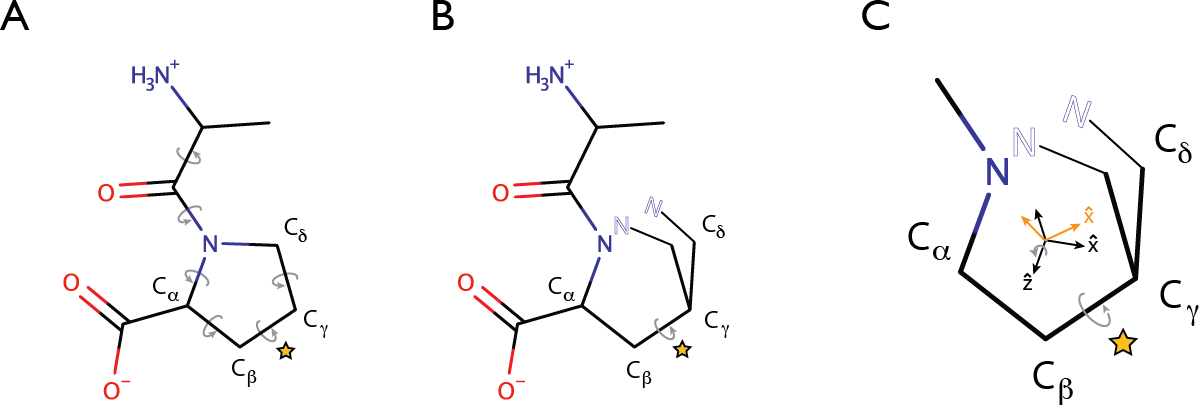
The split coordinate frame problem. **A.** Cycles in molecular topology complicate a tree-based description of molecular topology. Proline residues contain a cycle, as the side chain emanating from the *C*_*α*_ atom forms a closed ring, reentering the main chain at the N atom. Because of the requirement for ring closure, the torsion values (indicated by gray arrows) are not independent. **B.** The strategy for handling cycles in the Rosetta program is to create an artificial break in the ring, restoring a tree-like topology. The requirement for closure is enforced with the introduction of a ‘virtual’ N atom (denoted by the hollow typeface). Supplemental constraints are added to the energy potential to ensure that the virtual atom overlays its real atom counterpart. **C.** Interaction between ring breakage and local coordinate definitions complicate gradient calculation. A coordinate system is defined for each atom to properly orient its dipole and quadrupole moments in the global frame. This local frame is defined for each atom in terms of its bonded neighbors. For the N atom of proline, the z-axis is defined to lie along its bond with the *C*_*α*_ atom, and the x-axis lies in the plane formed by the z-axis and the direction towards the *C*_*δ*_ atom. When the bond between the N and *C*_*δ*_ atoms is artificially broken to reestablish a tree-like topology, rotations such as that about the torsion indicated by the star can lead to alteration of the local coordinate frame at the N atom (the two configurations of the *C*_*δ*_ atom give rise to the two local coordinate frames shown inside the proline ring). This change in local frame effects the interactions between the N atom and every other atom in the molecule, resulting in an energy gradient between atoms that are on the same side of a rotatable bond. Strong constraints to enforce the overlap between the ‘real’ and ‘virtual’ copies of the N atom can minimize the value of this gradient. However, this effect represents a violation of the original assumption of Noguti and Gō that energy potentials depend only on interatomic distances [31].

A common challenge for structural representations using torsional degrees of freedom is coping with cycles in the molecular topology. The situation is illustrated for an alanine-proline dipeptide in Fig 3. Assuming that the N-terminus is taken to be the root of the topological tree, the position of the proline nitrogen is determined by the torsion bond between the *C*_*α*_ and *C* atoms of the alanine residue. However, the proline side chain contains a flexible cycle that connects the *C*_*α*_ and N atoms of the residue. Consequently, the torsion bonds around the cycle are not independent (Fig 3A). The incompatibility between such cycles and the tree-based topologies required for efficient processing of macromolecular structure is well-known [36]. One way to handle flexible rings is through the introduction of virtual atoms. This is illustrated in Fig 3B, where the ring topology of the proline side chain has been artificially broken, and the side chain instead terminates in a virtual atom (indicated by an ‘N’ shown in outline) whose position is compared to the real N atom of the proline residue. In the Rosetta program, a harmonic restraint serves to penalize any deviation from co-locality between the virtual and the real atom. The location of this unphysical atom is determined by the torsional degrees of freedom in the side chain, but the only energy terms in which the virtual atom participates involve comparisons between its own position and that of its corresponding real atom.

The unphysical breaking of rings in the treatment of macromolecular topology has an undesirable effect on orientation-dependent gradient calculations such as those required for multipole electrostatics. The dipole and quadrupole moments for each atom are defined in a local coordinate system, which is defined in terms of the atom’s bonded neighbors. For some atom types, the neighbors required to define the coordinate system may lie across a break in the molecular topology. For instance, in the Amoeba force field, the coordinate system for the N atom in proline is defined in terms of the *C*_*α*_ and *C*_*δ*_ atoms. The *C*_*δ*_ atom lies across a ring break from the N atom in the chain topology. Rotations about the torsion bond indicated by a star in Fig 3C result in movements of the *C*_*δ*_ atom, and thus cause a change in the local coordinate system for the N atom. The change in local coordinate system alters the orientation of the dipole and quadrupole moments of the N atom, and thus its interactions with all other atoms in the macromolecule, regardless of whether the atoms lie on the same side of the rotating torsion bond. The only atom types for which this problem manifests in the Rosetta program are the proline N atom and cystine SG atoms in a disulfide bond. In theory, the local coordinate systems for these atoms could be redefined using only bonded neighbors on one side of the break in ring topology. For the purpose of demonstration, however, we wished to retain the exact parameters and functional form of the Amoeba force field to enable faithful comparisons. In practice, the strong harmonic potential constraining the location of the virtual atom to that of the real atom minimizes the error due to this effect, particularly during the end stages of molecular optimization.

### Comparison to numerical approximation

We have implemented the polarizable multipole electrostatics potential from the Amoeba force field in the Rosetta molecular modeling program, and verified that the values calculated for the potential match those obtained from the Tinker implementation of the Amoeba force field for a test set of proteins (Figure A in S1 Appendix). Derivative calculations are implemented as described above, both with and without the treatment of reference atoms necessary for handling frame rotation effects. To verify that the calculated derivatives are correct, we have compared the analytically computed values of the gradient to finite difference approximations obtained by evaluating the electrostatics potential for macromolecular conformations in which each torsional degree of freedom has been rotated by +/- 1.0x10^-3^ degrees. We performed this comparison for every rotatable bond in a test set of high-resolution crystal structures (63,344 total torsional degrees of freedom across 107 structures.)

**Fig 4:**
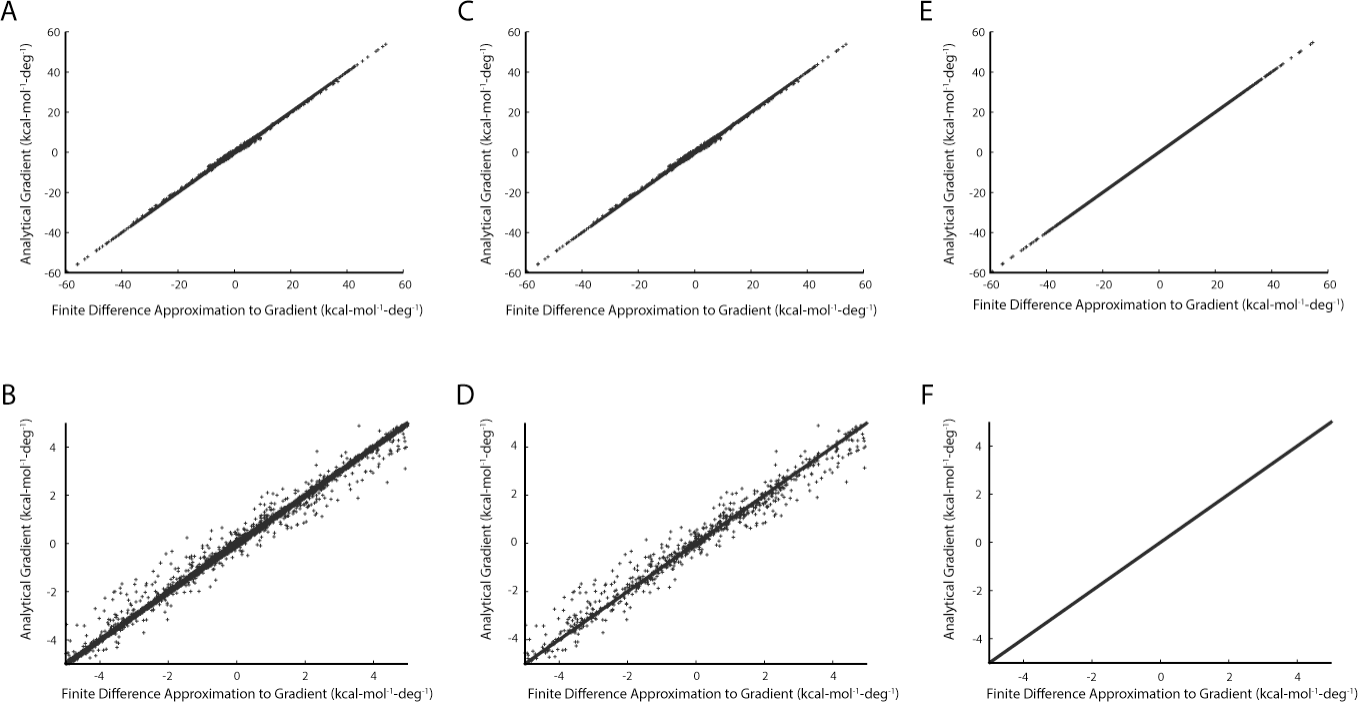
Verification of correctness for multipole electrostatic gradients. **A.** Analytical gradients were calculated for every torsional degree of freedom in a test set of proteins (see Methods) without incorporating reference atoms to address the frame rotation problem. In addition, a numerical approximation to the gradient was calculated by evaluating the electrostatic energy for conformations in which each degree of freedom was perturbed by +/- 1.0x10^-3^ degrees, in turn. **B.** The same data is shown for the restricted range of -5 to 5 kcal-mol^-1^-deg^-1^, within which ~77% of the data points fall. **C., D.** Similar data to panels A and B are shown, with the difference that the analytical gradients now include the reference atom treatment to account for frame rotation. **E., F.** Similar data to panels C and D, in which the dipole and quadrupole parameters for the two atom types that suffer from the split frame problem have been artificially zeroed out.

The results are shown in Fig 4. Comparison of the data from calculations without (panels 4A and 4B) and with (panels 4C and 4D) the reference atom treatment of frame rotation effects shows the improvement due to this correction. While a sparse cloud of off-diagonal points can be seen in both panels 4B and 4D, the line of points that falls roughly on the diagonal has become narrower with the reference atom treatment. The average absolute difference between the analytical and approximate gradients improves from 3.0x10^-2^ kcal-mol^-1^-deg^-1^ to 8.0x10^-3^ kcal-mol^-1^-deg^-1^. We speculated that the remaining off-diagonal scatter was due to the split frame problem. To test whether this was the case, we repeated the calculations with the multipole moments set to zero for the two atom types that exhibit this problem (proline N and disulfide SG). The data are shown in panels 4E and 4F. The scatter has indeed been eliminated, and the average absolute difference between the analytical and approximate gradients plunges to 1.2x10^-7^ kcal-mol^-1^-deg^-1^. This demonstrates that the use of reference atoms solves the frame rotation problem. We repeated this analysis with the perturbation for the finite-difference approximation increased by factors of two and ten. As is expected, the differences between the analytical and approximate gradients become slightly larger (Table A in S1 Appendix), but the results are independent of perturbation size.

We next looked at the contribution to the gradient at each torsional degree of freedom due to all possible atom-atom interactions in a single structure. The structure we used for this analysis (chain B of pdb code 4K12) has 84 residues and 1352 atoms (once hydrogen atoms have been added). This structure contains no disulfide bonds or proline residues, and therefore no examples of the split frame problem. There are 441 torsional degrees of freedom, giving a total of over 400 million possible atom pair/torsion bond combinations. However, when the atoms in a pair do not lie on opposite sides of the degree of freedom, they do not contribute to the energy gradient for that torsion. For this structure, there were over 86 million non-zero atom pair/torsion combinations. For each combination we compared the analytically calculated gradient with a finite-difference approximation (see Methods) (Figure B in S1 Appendix). The average absolute difference for non-zero gradients at this fine-grained level was very small (3.6x10^-7^ kcal-mol^-1^-deg^-1^). Of the over 86 million gradients in our analysis, only 396 had differences greater than 1.0x10^-3^ kcal-mol^-1^-deg^-1^. The largest observed difference was 1.3x10^-3^ kcal-mol^-1^-deg^-1^, which represented a difference of 1.3% for that particular atom pair/torsion combination. These results indicate that the agreement we observed at the level of summed analytical and approximate gradients for each degree of freedom is not the result of cancellation of larger errors at the level of individual atom pairs.

### Derivatives of solvent accessible surface area in torsional framework

Implicit models for the effects of water on macromolecular energetics typically include contributions from the damping of electrostatics by the bulk dielectric influence of water as well as from the cost of cavity formation estimated from the molecular solvent accessible surface area (SASA). The Ponder lab has reported an extension to the Amoeba force field called the generalized Kirkwood method that includes the effects of solvent as a bulk dielectric upon multipole electrostatics [30]. The relevant modifications to energy and gradient calculations are straightforward to incorporate, and require no further derivatives beyond those given above. We now present the derivatives for the SASA with respect to the torsional bonds in a macromolecule to model the cavity formation component of solvation.

**Fig 5:**
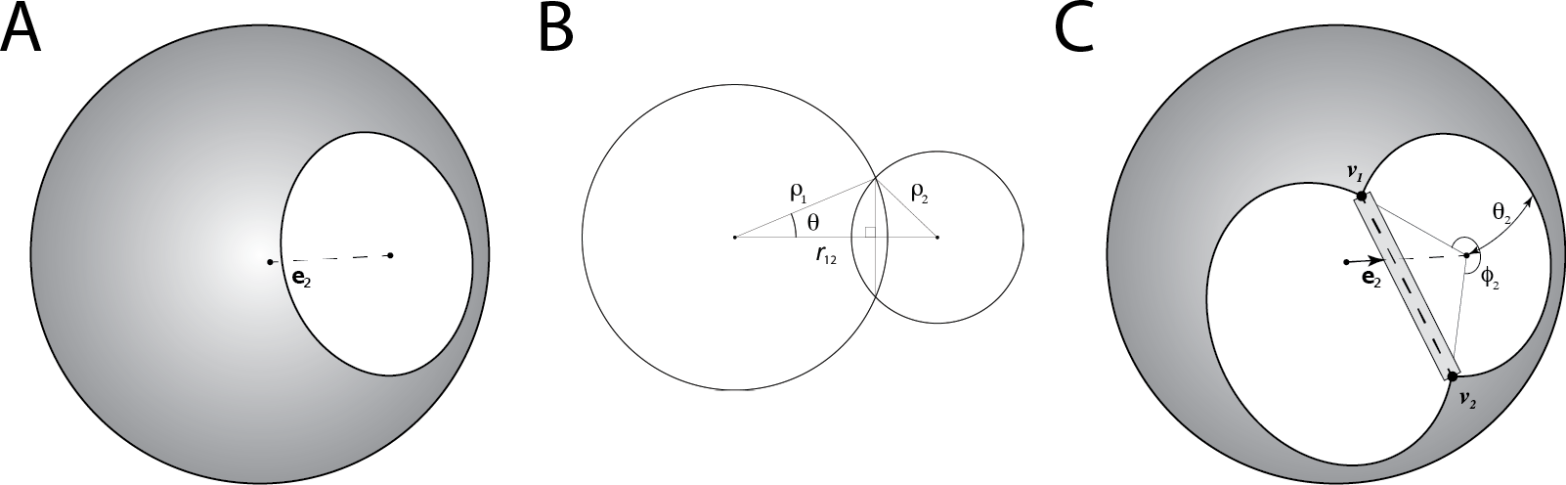
Evaluating SASA derivatives from intersectional information. Expressions for the derivative of SASA of a molecule with respect to its torsional degrees of freedom can be deduced from the arcs that define the intersections of spherical overlaps, without differentiating the full expression for each atomic SASA. **A.** An atom is shown rendered as a grey sphere with a patch buried by a neighboring atom colored in white. The unit vector ***e*** shows the direction from the atom under consideration to the neighboring atom responsible for the overlap. The first contribution to the SASA derivative comes from motions that change the distance between two atoms. In the absence of other atoms, the rotation of the neighboring atom about the atom of interest serves only to relocate the buried patch without changing the SASA. **B.** The change in the size of the buried patch due to another atom with respect to interatomic separation can be determined by applying the law of cosines to the intersectional geometry for the spheres. **C.** When multiple patches overlap, the surface of the sphere that is occluded is described by a set of arcs. In this case, there are two arcs. One arc goes clockwise from ***v***_**1**_ to ***v***_**2**_, and a second arc completes the cycle clockwise from ***v***_**2**_ to ***v***_**1**_. The contribution to the change in SASA due to altered distance to a neighboring atom is modified relative to the two atom case in that only a fraction of the buried patch is independent of other atoms. In this example, the change in patch size with respect to interatomic separation in panel A is scaled by $\frac{\varphi_{2}}{2\pi }$. In addition, a second contribution to the SASA derivative results from distance-preserving rotations of intersecting atoms about the atom under consideration. Infinitesimal rotations perpendicular to the line between the points that define each arc (***v***_**1**_ and ***v***_**2**_ in panel C) sweep out an infinitesimal slice of surface area (shown as a grey box).

Different approaches have been described for calculating the exact SASA of a macromolecule [37–42]. Each ultimately obtains cycles of arcs that circumscribe the exposed patches of the SASA for each atom. The SASA is then calculated from each cycle using the Connolly formula [37]. We have implemented the power diagram based method due to Klenin, et. al. in Rosetta for obtaining these cycles [38], but many algorithms are suitable for this purpose. When calculating gradients, it has been noted previously that it is not necessary to differentiate the Connolly formula with respect to atomic coordinates to obtain derivatives for the atomic SASA[38,43]. Rather, the SASA derivative can be deduced directly from the effect that the movement of an atom has on the area of the cap buried between itself and a neighboring atom. This effect has two contributions. The first is due to parallel displacement, in which one atom moves either towards or away from a neighboring atom without rotation about the neighboring atom’s center (Fig 5A and 5B). The second contribution comes from perpendicular displacement, where an atom maintains a constant distance from a neighboring atom, but instead moves in a direction perpendicular to the line connecting the atoms’ centers (Fig 5C).

For a pair of atoms 1, 2 that share one or more arcs on the SASA on a molecule (for example, the clockwise arc from ***v***_**1**_ to ***v***_**2**_ in Fig 5C), the change in SASA due to the displacement of atom 2 relative to atom 1 is given by [38,43]:

$$\frac{\partial SASA}{\partial r_{12}}=\sum_{arcsi}\left[\frac{\phi_{i}\rho_{1}}{2r_{12}}\left(1-\frac{\rho_{1}^{2}-\rho_{2}^{2}}{r_{12}^{2}}\right)r_{12}-\frac{\rho_{1}}{r_{12}^{2}}\left(\left(\upsilon_{i+1}-\upsilon_{i}\right)\times r_{12}\right)\right].$$ (14)

The first term in the brackets accounts for the parallel displacement contribution. The derivative of the SASA with respect to the distance between the atoms is calculated using the law of cosines, and the resulting value is scaled by the fraction of the full circle of intersection between the atoms that is represented by the arc ($\frac{\varphi_{i}}{2\pi }$; see Fig 5C). The second term accounts for perpendicular displacement, which can be thought of as the change in SASA due to movement of a circle of intersection between two atoms either towards or away from other circles of intersections at the terminal points of a given arc. For an arc terminated at points ***v***_**1**_ and ***v***_**2**_, only motion that is perpendicular to the line between the two points results in a change in SASA. For the situation in Fig 5C, movement of the circle of intersection on the right-hand side orthogonal to the line connecting points ***v***_**1**_ and ***v***_**2**_ (shown as a dashed line) sweeps out an infinitesimal slice of surface area (denoted in gray).

To express these derivatives in a suitable form for the framework of Gō and coworkers, we take the dot product of (14) with the expression for $\frac{\partial r_{12}}{\partial \alpha }$ given in (9) to obtain the following expression:

$$\frac{\partial SASA}{\partial r_{12}}∙\frac{\partial r_{12}}{d\alpha }=-\Phi_{∥}r_{12}∙(n_{\alpha }\times r_{2})+\Phi_{∥}r_{12}∙(n_{\alpha }\times r_{\alpha })+\Phi_{⊥}V∙(n_{\alpha }\times r_{2})-\Phi_{⊥}V∙(n_{\alpha }\times r_{\alpha })$$ (15)

where we have introduced the following simplifying definitions:

$$\Phi_{∥}\equiv \sum_{arcsi}\frac{\phi_{i}\rho_{1}}{2r_{12}}(1-\frac{\rho_{1}^{2}-\rho_{2}^{2}}{r_{12}^{2}})$$ (16)

$$\Phi_{⊥}\equiv \frac{\rho_{1}}{r_{12}^{2}}$$ (17)

$$V\equiv \sum_{arcsi}(\upsilon_{i+1}-\upsilon_{i})\times r_{12}$$ (18)

Following our approach above, we rewrite vector products so that every term involves a dot product with either ***n***_*α*_ or (***n***_*α*_ × ***r***_*α*_), and group these terms to obtain:

$$\frac{\partial SASA}{\partial r_{12}}∙\frac{\partial r_{12}}{d\alpha }=-[\Phi_{∥}∙(r_{2}\times r_{12})+\Phi_{⊥}(r_{2}\times V)]∙n_{\alpha }+[\Phi_{∥}r_{12}+\Phi_{⊥}V]∙(n_{\alpha }\times r_{\alpha })$$ (19)

Here we can identify the vectors ***f*** and ***g*** required in the Gō framework:

$$f=-[\Phi_{∥}∙(r_{2}\times r_{12})+\Phi_{⊥}(r_{2}\times V)]$$ (20)

$$g=[\Phi_{∥}r_{12}+\Phi_{⊥}V].$$ (21)

### Verification of correctness for SASA derivatives

**Fig 6:**
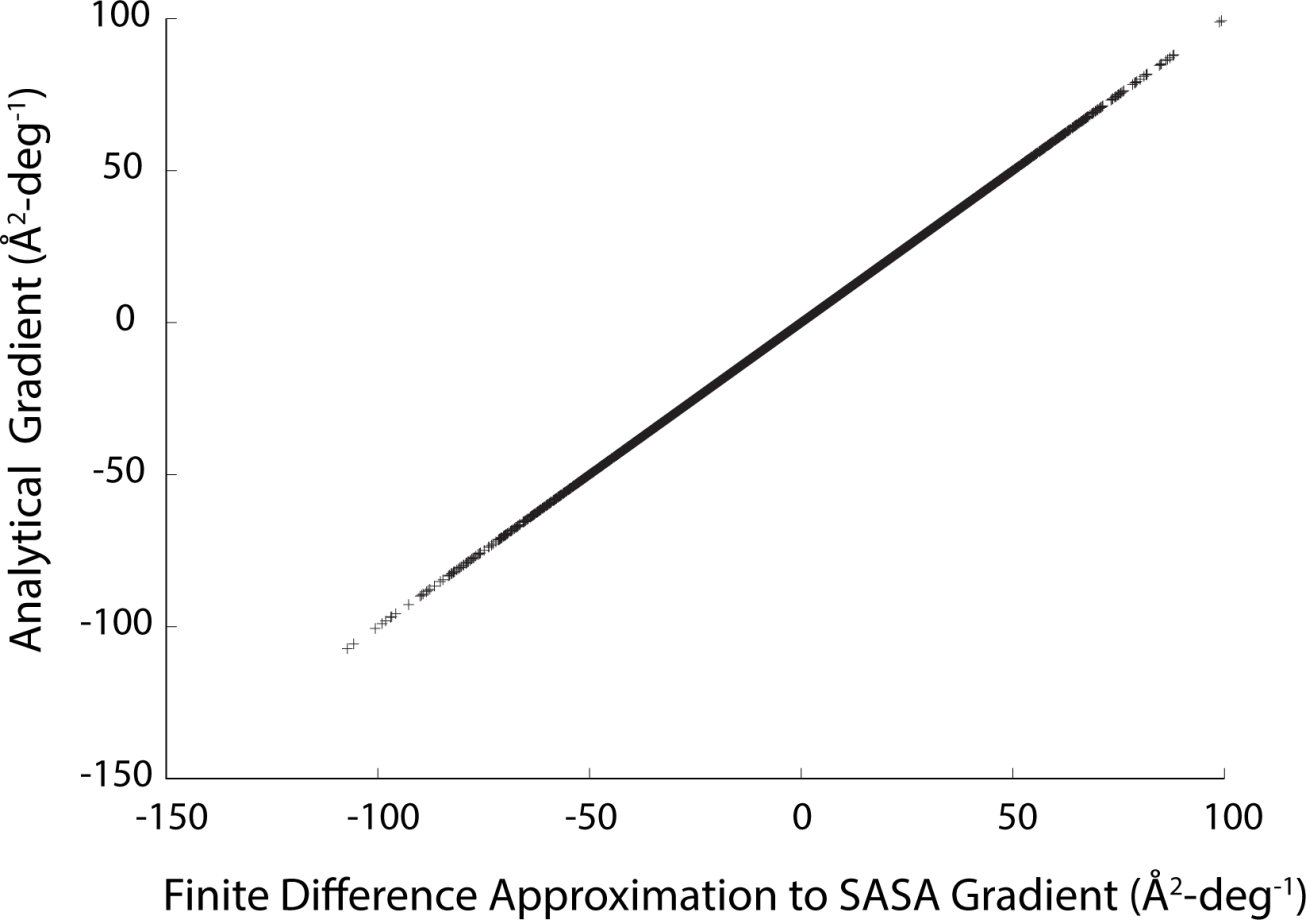
Verification of correctness for SASA gradients. Analytical gradients for the SASA values of a test set of protein structures were calculated with respect to all torsional bonds in the structures. For each torsion bond, a numerical approximation to the gradient was calculated by evaluating the SASA for perturbed structures in which each degree of freedom was varied by +/- 1.0x10^-3^ degrees.

We have implemented the power diagram algorithm of Klenin et. al. to obtain the arc information necessary to compute molecular SASA in the Rosetta program [38]. The derivatives in the previous section were used to compute the gradients of a surface area-scaled potential energy term with respect to rotatable bonds in a macromolecule. As before, we verified the correctness of the derivatives by comparing the analytically computed values of the gradient to finite difference approximations obtained by comparing SASA calculations after perturbing torsion bonds by small amounts. We used the same test set as before for this evaluation (63,344 total torsional degrees of freedom across 107 high-resolution crystal structures.) The results are shown in Fig 6. Agreement is essentially exact for all rotatable degrees of freedom in the test set (average absolute error of 6.4x10^-5^ Å^2^-deg^-1^), indicating that we have obtained suitable derivatives of SASA for use in torsional systems. When the perturbation applied to the torsional bonds is doubled, the results are similar, but when it is increased by an order of magnitude, the agreement with the analytical value deteriorates (Table A in S1 Appendix). This is a consequence of the short-range, discontinuous nature of atomic overlap. Larger bond rotations can cause overlapping atoms to separate and separated atoms to overlap. In some cases this may lead line minimization algorithms to take smaller descent steps to ensure that the system does not move outside the region of validity for the calculated gradient.

To obtain a finer-grained assessment, we next examined the contribution of the changes in the SASA for every atom in a structure to the derivatives at each torsional degree of freedom. We performed this detailed analysis on five structures (PDB codes: 1EAQA chain A, 1XODA chain A, 2Z5W chain A, 4K12 chain A, and 5COFA chain A). For these five structures, there were over 1.1 million atomic SASA/torsion combinations. For each combination we compared the analytically calculated gradient with a finite-difference approximation (see Methods) (Figure C in S1 Appendix). The average absolute difference between the analytical and approximate gradients was very small (2.8x10^-7^ Å^2^-deg^-1^). These results lend further support to the conclusion that our equations for the derivatives of atomic SASA with respect to torsional degrees of freedom are correct.

## Discussion

We have presented the derivatives for multipole electrostatics and solvent-accessible surface area in the efficient recursive framework for calculating gradients in a torsional system developed by Gō and coworkers. In the interest of enabling a complete, implicit treatment of solvation, we have implemented the Amoeba polarizable multipole potential with the generalized Kirkwood model, and a SASA based cavity formation penalty in the Rosetta molecular modeling program. The analytically calculated gradients have been validated by comparison to finite difference approximation, indicating the correctness of our derivations.

The framework for rapidly calculating and accumulating gradient information for minimization in systems with torsional degrees of freedom presented by Gō and coworkers was limited to potential terms that depended on either atomic distances alone, or were simple functions of the torsion angles at the degrees of freedom themselves. Subsequent work has extended the applicability to potentials that depend on angles and torsions between arbitrary atoms [35]. We have presented a technique for further incorporating orientation-dependent potentials, for which the interaction energies between atoms can change even if the interatomic distances do not. We have accomplished this using the notion of ‘reference atoms’, which incorporate orientational effects while preserving the overall logic of the original recursive gradient accumulation algorithm. We have also presented for the first time an approach to handling potentials with terms that include tensor derivatives with respect to torsion bonds (constant tensor terms had been previously treated [35]). These general approaches to computing gradients for complex potentials in torsional systems should prove useful for development of potentials in the future.

The derivatives for solvent accessible surface area with respect to rotational degrees of freedom do not depend on the orientations of the atoms in a macromolecular system, as they are modeled as spheres. The only derivative required is that of the displacement vector between two atoms. Building on the observations of others that only certain relative displacements of atoms contribute to changes in exposed surface patches (namely, displacements along the vector between two atoms and distance-preserving movement perpendicular to the endpoints of exposed arcs), we find that these two contributions are easily extracted from the derivatives of displacement vectors with respect to torsional rotations by projecting the changes in displacement onto these directions.

The benefits of polarizable multipole models for electrostatics come at significant computational cost. The full set of potential terms through the quadrupole interactions is comprised of 14 individual energy terms. Incorporating polarization only adds to the burden, as does an implicit treatment of the effects of solvent on electrostatics. The decision to model cavity-formation in a solvent with a surface area-scaled energy term also incurs a significant cost: in our case, we must construct a power diagram for our macromolecule to obtain the arcs that define patches of accessible surface area whenever derivatives are required. Whether the additional accuracy provided is worth the cost, or whether more extensive conformational sampling with a simpler potential is a better investment of computational resources, is an open question.

By implementing the Amoeba electrostatics model in Rosetta, we hope that the answer to this question can be pursued in a diverse set of applications for which the Rosetta program has proven useful, such as protein-protein docking [32,44], enzyme design [45,46], and the modeling of protein-DNA interfaces [47,48]. The Amoeba electrostatics potential involves parameters for monopole, dipole, and quadrupole moments as well as polarizability for each atom in a simulation. While parameters are available for canonical amino and nucleic acids, they are not generally available for small molecules that may be of interest in protein-ligand docking. However, software has been developed to obtain these parameters automatically [49]. As a demonstration that multipole electrostatics can be readily combined with the capabilities of the Rosetta program, we have minimized a ternary complex of a lambda repressor dimer with operator DNA under a variant of the Rosetta energy function incorporating polarizable multipole electrostatics and a SASA-scaled treatment of cavity formation (Figure D in S1 Appendix).

## Supporting information

S1 Appendix Sample derivation of multipole gradient term, figures of fine-grained derivative comparison, and example minimization of protein-DNA complex using multipole electrostatic energy and solvent-accessible surface area scaled potential terms. (DOCX)
