# Supplementary material for: Efficient minimization of multipole electrostatic potentials in torsion space
Source: PLoS One. 2018 Apr 11;13(4):e0195578. doi: 10.1371/journal.pone.0195578 (PMC5895050; doi:10.1371/journal.pone.0195578)
Supplement: S1 Appendix — (DOCX) [file pone.0195578.s001.docx]

**Supplemental Materials**

**Example calculation of multipole term derivative**

The dipole‑quadrupole interaction between two atoms is described by several terms (see Fig 2 in the manuscript), one of which is:

$E_{12}=\frac{2\left( \boldsymbol{r}_{12}\cdot\boldsymbol{\Theta}_{1}\cdot\boldsymbol{\mu}_{2} \right)}{r_{12}^{5}}$ (S1).

We desire the derivative of this term with respect to an arbitrary torsion α. By convention, we take regions upstream of the torsion bond α to be fixed, meaning that the rotation moves only the second atom. As a result, there are no derivatives with respect to the multipoles of the first atom. Expanding the derivative with the chain rule for each independent variable gives:

$\frac{dE_{12}}{d\alpha}=\frac{\partial E_{12}}{\partial r_{12}}\frac{\partial r_{12}}{\partial\alpha}+\frac{\partial E_{12}}{\partial\boldsymbol{r}_{\boldsymbol{12}}}\cdot\frac{\partial\boldsymbol{r}_{\boldsymbol{12}}}{\partial\alpha}+\frac{\partial E_{12}}{\partial\boldsymbol{\mu}_{\boldsymbol{2}}}\cdot\frac{\partial\boldsymbol{\mu}_{\boldsymbol{2}}}{\partial\alpha}$ (S2).

The partial derivatives for the energy with respect to the distance between the atoms, the vector between the atoms, and the dipole moment are readily obtained by differentiating equation S1 with respect to each variable.

$\frac{\partial E_{12}}{\partial r_{12}}=\frac{-10\left( \boldsymbol{r}_{12}\cdot\boldsymbol{\Theta}_{1}\cdot\boldsymbol{\mu}_{2} \right)}{r_{12}^{7}}$ (S3)

$\frac{\partial E_{12}}{\partial\boldsymbol{r}_{\boldsymbol{12}}}=\frac{2}{r_{12}^{5}}\left( \boldsymbol{\Theta}_{1}\cdot\boldsymbol{\mu}_{2} \right)$ (S4)

$\frac{\partial E_{12}}{\partial\boldsymbol{\mu}_{\boldsymbol{2}}}=\frac{2}{r_{12}^{5}}\left( \boldsymbol{r}_{12}\cdot\boldsymbol{\Theta}_{1} \right)$ (S5)

The required partial derivatives for each of the independent variables with respect to the infinitesimal rotation α about an arbitrary axis are:

$\frac{\partial\boldsymbol{r}_{\boldsymbol{12}}}{\partial\alpha}=-\left( \boldsymbol{n}_{\boldsymbol{\alpha}}\boldsymbol{\times}\boldsymbol{r}_{\boldsymbol{2}}\boldsymbol{-}\boldsymbol{n}_{\boldsymbol{\alpha}}\boldsymbol{\times}\boldsymbol{r}_{\boldsymbol{\alpha}} \right)$ (S6)

$\frac{\partial r_{12}}{\partial\alpha}=\frac{\partial\sqrt{\boldsymbol{r}_{12}\cdot\boldsymbol{r}_{12}}}{\partial\alpha}= \frac{1}{r_{12}}\left[ \left( \boldsymbol{r}_{2}\times\boldsymbol{r}_{1} \right)\cdot\boldsymbol{n}_{\alpha}+ \boldsymbol{r}_{12}\cdot\left( \boldsymbol{n}_{\alpha}\times\boldsymbol{r}_{\alpha} \right) \right]$ (S7)

$\frac{\partial\boldsymbol{\mu}_{\boldsymbol{2}}}{\partial\alpha}=-\boldsymbol{n}_{\alpha}\times\boldsymbol{\mu}_{\boldsymbol{2}}$ (S8)

Here $\boldsymbol{n}_{\alpha}$ is the normal vector of the axis of rotation for the torsion bond, and $\boldsymbol{r}_{\boldsymbol{\alpha}}$ is the vector from the origin to the downstream atom defining the torsion bond. Combining equations S3‑5 with S6-8 gives:

$\frac{dE_{12}}{d\alpha}=\frac{-10\left( \boldsymbol{r}_{12}\cdot\boldsymbol{\Theta}_{1}\cdot\boldsymbol{\mu}_{2} \right)}{r_{12}^{7}}\left[ \left( \boldsymbol{r}_{2}\times\boldsymbol{r}_{1} \right)\cdot\boldsymbol{n}_{\alpha}+ \boldsymbol{r}_{12}\cdot\left( \boldsymbol{n}_{\alpha}\times\boldsymbol{r}_{\alpha} \right) \right]-\frac{2}{r_{12}^{5}}\left( \boldsymbol{\Theta}_{1}\cdot\boldsymbol{\mu}_{2} \right)\cdot\left( \boldsymbol{n}_{\boldsymbol{\alpha}}\boldsymbol{\times}\boldsymbol{r}_{\boldsymbol{2}}\boldsymbol{-}\boldsymbol{n}_{\boldsymbol{\alpha}}\boldsymbol{\times}\boldsymbol{r}_{\boldsymbol{\alpha}} \right)-\frac{2}{r_{12}^{5}}\left( \boldsymbol{r}_{12}\cdot\boldsymbol{\Theta}_{1} \right)\cdot\left( \boldsymbol{n}_{\alpha}\times\boldsymbol{\mu}_{\boldsymbol{2}} \right)$ (S9)

To clarify the equation we introduce the auxiliary vectors $\boldsymbol{I}_{\boldsymbol{1}}\boldsymbol{=}\boldsymbol{\Theta}_{\boldsymbol{1}}\cdot\boldsymbol{r}_{\boldsymbol{12}}$ and $\boldsymbol{J}_{\boldsymbol{1}}\boldsymbol{=}\boldsymbol{\Theta}_{\boldsymbol{1}}\cdot\boldsymbol{\mu}_{\boldsymbol{2}}$ .

$\frac{dE_{12}}{d\alpha}=\frac{-10\left( \boldsymbol{r}_{12}\cdot\mathbf{I}_{1} \right)}{r_{12}^{7}}\left[ \left( \boldsymbol{r}_{2}\times\boldsymbol{r}_{1} \right)\cdot\boldsymbol{n}_{\alpha}+ \boldsymbol{r}_{12}\cdot\left( \boldsymbol{n}_{\alpha}\times\boldsymbol{r}_{\alpha} \right) \right]-\frac{2}{r_{12}^{5}}\mathbf{J}_{1}\cdot\left( \boldsymbol{n}_{\boldsymbol{\alpha}}\boldsymbol{\times}\boldsymbol{r}_{\boldsymbol{2}}\boldsymbol{-}\boldsymbol{n}_{\boldsymbol{\alpha}}\boldsymbol{\times}\boldsymbol{r}_{\boldsymbol{\alpha}} \right)-\frac{2}{r_{12}^{5}}\mathbf{I}_{1}\cdot\left( \boldsymbol{n}_{\alpha}\times\boldsymbol{\mu}_{\boldsymbol{2}} \right)$ (S10)

In order to conform to the Noguti and Gō treatment in which all derivatives must be written in the form

$\frac{{\partial E}_{i,j}}{\partial\theta_{\alpha}}=\boldsymbol{f}_{i,j}\boldsymbol{\cdot}\boldsymbol{n}_{\alpha}\boldsymbol{+}\boldsymbol{g}_{i,j}\boldsymbol{\cdot}\left( \boldsymbol{n}_{\alpha}\boldsymbol{\times}\boldsymbol{r}_{\alpha} \right)$**.** (S11)

We make use of our ability to cyclically permute vector triple cross products to bring the expression for the derivative closer to the desired form:

$\frac{dE_{12}}{d\alpha}=\frac{-10\left( \boldsymbol{r}_{12}\cdot\mathbf{I}_{1} \right)}{r_{12}^{7}}\left[ \left( \boldsymbol{r}_{2}\times\boldsymbol{r}_{1} \right)\cdot\boldsymbol{n}_{\alpha}+ \boldsymbol{r}_{12}\cdot\left( \boldsymbol{n}_{\alpha}\times\boldsymbol{r}_{\alpha} \right) \right]-\frac{2}{r_{12}^{5}}\left( \boldsymbol{r}_{\boldsymbol{2}}\boldsymbol{\times}\mathbf{J}_{1} \right)\cdot\boldsymbol{n}_{\boldsymbol{\alpha}}+\frac{2}{r_{12}^{5}}\mathbf{J}_{1}\cdot\left( \boldsymbol{n}_{\boldsymbol{\alpha}}\boldsymbol{\times}\boldsymbol{r}_{\boldsymbol{\alpha}} \right)-\frac{2}{r_{12}^{5}}\left( \boldsymbol{\mu}_{\boldsymbol{2}}\times\mathbf{I}_{1} \right)\cdot\boldsymbol{n}_{\alpha}$ (S12)

By convention we place the $\boldsymbol{I}_{\boldsymbol{1}}$ and $\boldsymbol{J}_{\boldsymbol{1}}$ vectors in the first position of cross products, yielding the final form:

$\frac{dE_{12}}{d\alpha}=\frac{-10\left( \boldsymbol{r}_{12}\cdot\mathbf{I}_{1} \right)}{r_{12}^{7}}\left[ \left( \boldsymbol{r}_{2}\times\boldsymbol{r}_{1} \right)\cdot\boldsymbol{n}_{\alpha}+ \boldsymbol{r}_{12}\cdot\left( \boldsymbol{n}_{\alpha}\times\boldsymbol{r}_{\alpha} \right) \right]+\frac{2}{r_{12}^{5}}\left( \mathbf{J}_{1}\boldsymbol{\times}\boldsymbol{r}_{\boldsymbol{2}} \right)\cdot\boldsymbol{n}_{\boldsymbol{\alpha}}+\frac{2}{r_{12}^{5}}\mathbf{J}_{1}\cdot\left( \boldsymbol{n}_{\boldsymbol{\alpha}}\boldsymbol{\times}\boldsymbol{r}_{\boldsymbol{\alpha}} \right)+\frac{2}{r_{12}^{5}}\left( \mathbf{I}_{1}\times\boldsymbol{\mu}_{\boldsymbol{2}} \right)\cdot\boldsymbol{n}_{\alpha}$ (S14)

By grouping the terms that involve dot products with $\boldsymbol{n}_{\alpha}$ and those with $\left( \boldsymbol{n}_{\alpha}\boldsymbol{\times}\boldsymbol{r}_{\alpha} \right)$ we can identify the required terms for the Noguti and Gō framework.

$\boldsymbol{f}=\frac{2}{r_{12}^{5}}\left( \boldsymbol{I}_{\boldsymbol{1}}\times\boldsymbol{\mu}_{\boldsymbol{2}} \right)-\frac{10\left( \boldsymbol{I}_{\boldsymbol{1}}\cdot\boldsymbol{\mu}_{\boldsymbol{2}} \right)}{r_{12}^{7}}\left( \boldsymbol{r}_{\boldsymbol{2}}\times\boldsymbol{r}_{\boldsymbol{1}} \right)+\frac{2}{r_{12}^{5}}\left( \boldsymbol{J}_{\boldsymbol{1}}\times\boldsymbol{r}_{\boldsymbol{2}} \right)$ (S15)

and

$\boldsymbol{g}=\frac{2}{r_{12}^{5}}\boldsymbol{J}_{\boldsymbol{1}}-\frac{10\left( \boldsymbol{I}_{\boldsymbol{1}}\cdot\boldsymbol{\mu}_{\boldsymbol{2}} \right)}{r_{12}^{7}}\boldsymbol{r}_{\boldsymbol{12}}$ (S16)

These values match the entries for the first quadrupole-dipole term of Table 1.

**Minimization of lambda repressor‑DNA complex**

Starting coordinates for the heavy atoms in the complex were taken from a 1.8 Å crystal structure (PDB code: 1LMB [1]). Hydrogens were placed using the Rosetta molecular modeling program. Energy minimization was accomplished with a BFGS Quasi-Newton algorithm using a Moré‑Thuente line minimizer [2]. The force field used for minimization was the ‘talaris2013’ scoring function , with the distance dependent dielectric electrostatics term, the geometry‑dependent hydrogen bonding term, and the Lazaridis‑Karplus solvation term removed [3]. These terms were replaced with the Amoeba polarizable multipole electrostatics potential augmented by the generalized Kirkwood implicit solvent electrostatics treatment [4] and a solvent-accessible surface area scaled cavity formation term. The ‘amoebabio09.prm’ parameters for the Amoeba force field were used, as they contain provisional values for nucleic acids [5]. The maximum number of iterations was set to 500, although this limit was not met.

The weights assigned to the component energy terms were:

fa_vdw_tinker 1.0

pro_close 1

rama 0.2

omega 0.5

fa_dun 0.56

p_aa_pp 0.32

multipole_elec 1.00

fa_sasa 0.005

dna_bb_torsion 0.60

dna_sugar_close 0.85

dna_chi 0.5

ref 1

No attempts were made to optimize the weights assigned to the newly introduced energy terms. Any production use of these new terms would require a full‑fledged weight optimization effort, which is outside the scope of this manuscript. **Table A**

| Size of perturbation  (deg) | Multipole term  (w/o reference atoms)  (kcal‑mol^‑1^‑deg^‑1^) | Multipole term  (with reference atoms)  (kcal‑mol^‑1^‑deg^‑1^) | Multipole term  (with N_pro_ and SG_disulfide_ moments zeroed out)  (kcal‑mol^‑1^‑deg^‑1^) | SASA term  (Å^2^‑deg^‑1^) |
| --- | --- | --- | --- | --- |
| 0.001 | 0.026 (abs)  0.35 (frac) | 0.0082 (abs)  0.043 (frac) | 1.2x10^-7^ (abs)  7.5x10^-8^ (frac) | 6.4x10^-5^ (abs)  1.4x10^-5^ (frac) |
| 0.002 | 0.030 (abs)  0.35 (frac) | 0.0082 (abs)  0.043 (frac) | 4.9x10^-7^ (abs)  2.6x10^-7^ (frac) | 1.9x10^-4^ (abs)  4.3x10^-5^ (frac) |
| 0.010 | 0.030 (abs)  0.35 (frac) | 0.0082 (abs)  0.043 (frac) | 1.2x10^-5^ (abs)  6.4x10^-6^ (frac) | 1.4 (abs)  5.6x10^-2^ (frac) |

**Figure A**


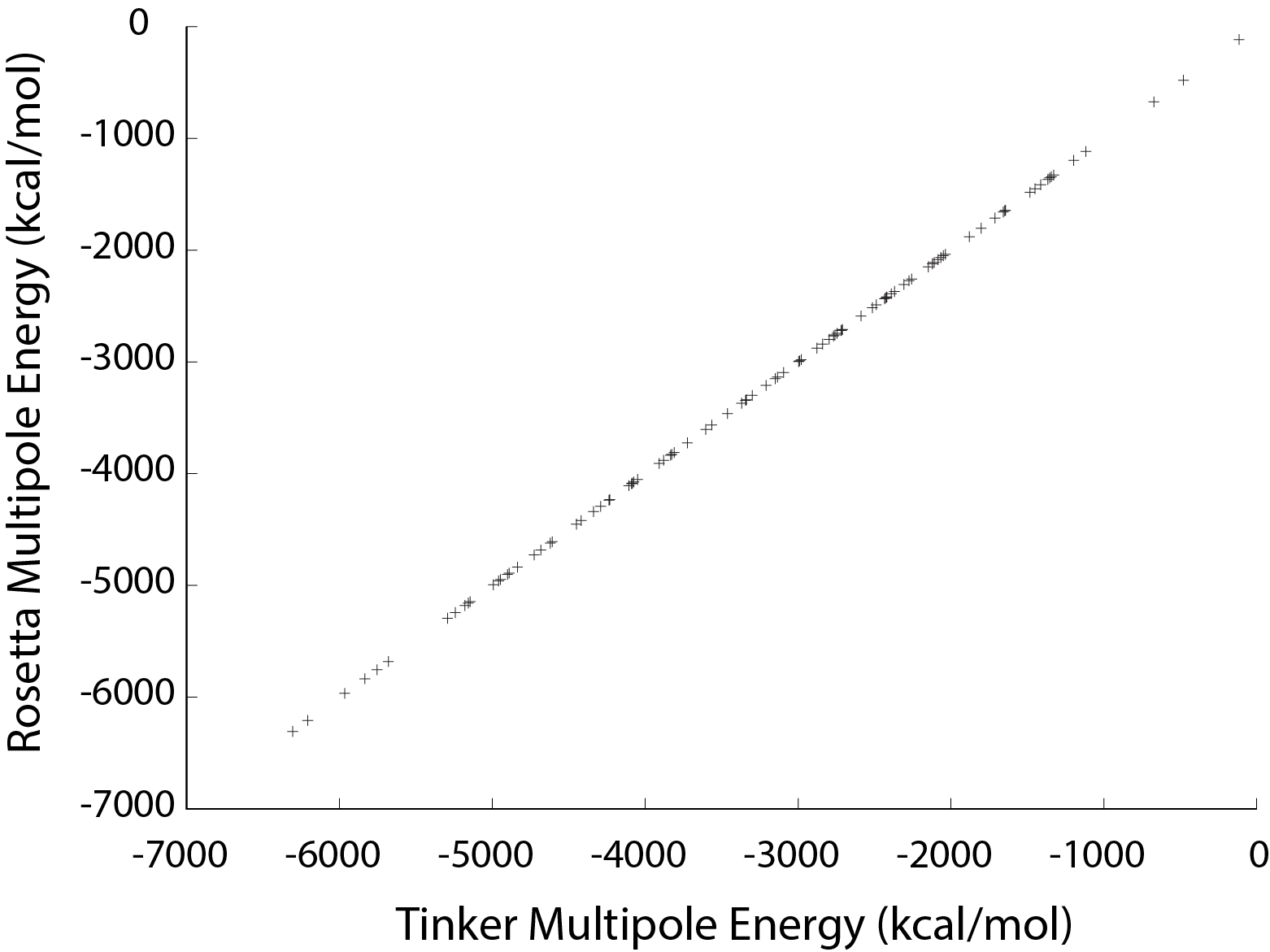


**Figure A. Comparison of multipole electrostatic energies calculated by the Tinker and Rosetta molecular modeling programs.** Multipole electrostatic energies were calculated for a set of protein structures (see Methods) using the Tinker and Rosetta modeling programs. The results are in close agreement. The maximum absolute difference between the energies calculated by the two programs for any structure is 0.19 kcal/mol (for a structure with an energy of -4050.12 kcal/mol as calculated by Tinker). The largest percentage difference for a structure is 0.01%.

**Figure B**


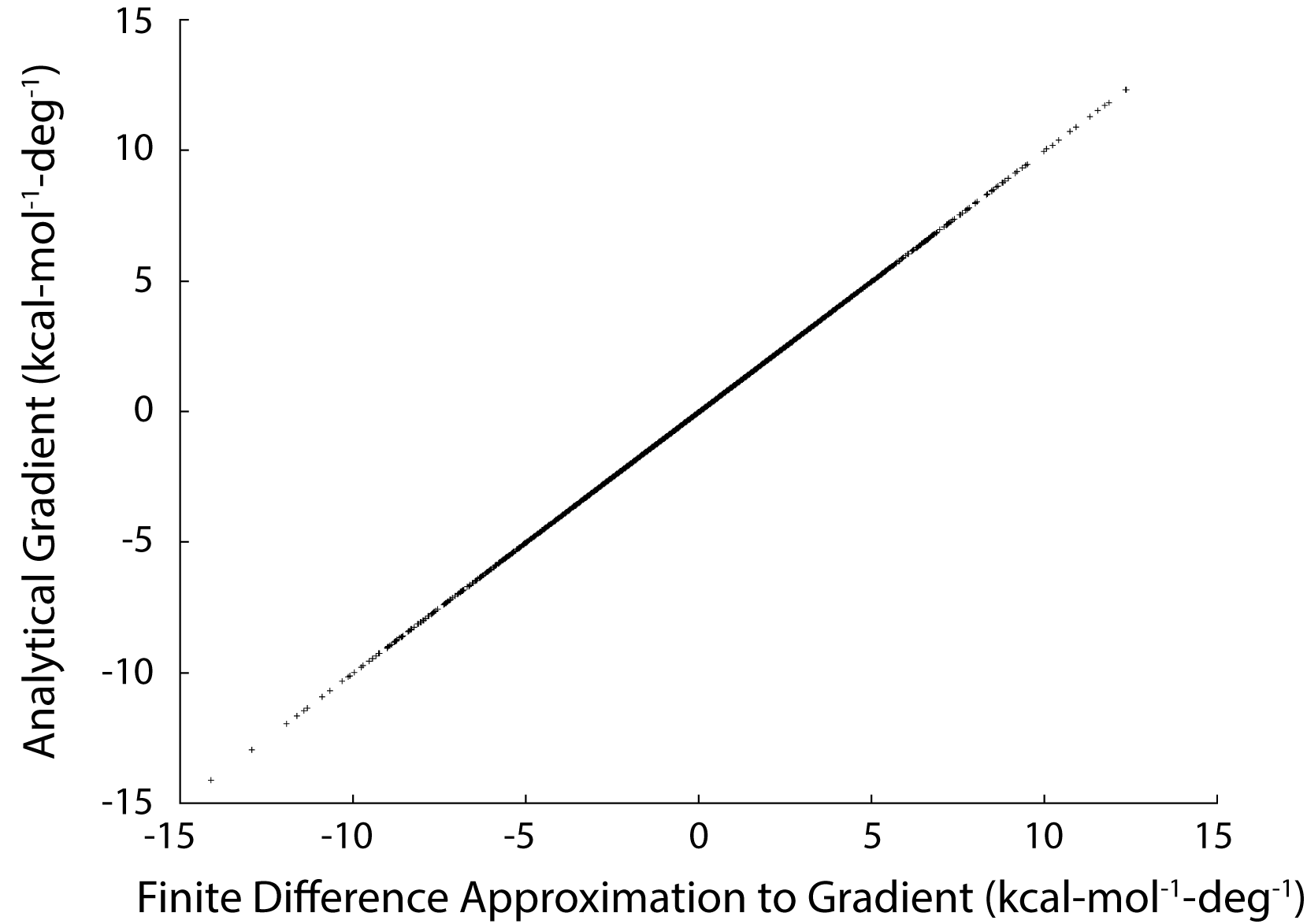


**Figure B. Comparison of contributions of atom‑atom pairs to individual torsion gradients calculated analytically with values obtained by finite‑difference approximation.** Analytically calculated and numerically approximated values for the contributions of all atom‑atom multipole interactions to the gradients at all torsional degrees of freedom in a single structure are compared. The structure (PDB code: 4K12, chain B) has 1352 atoms once hydrogen atoms are added to the heavy atoms in the PDB coordinate file, and 441 torsional degrees of freedom. Over 86 million combinations of atom pairs and torsion bonds have non‑zero gradients (rotation of the torsion bond results in a change in energy between the pair of atoms.) The average absolute difference between the analytical and approximate values is 3.6x10^-7^ kcal‑mol^‑1^‑deg^‑1^.

**Figure C**


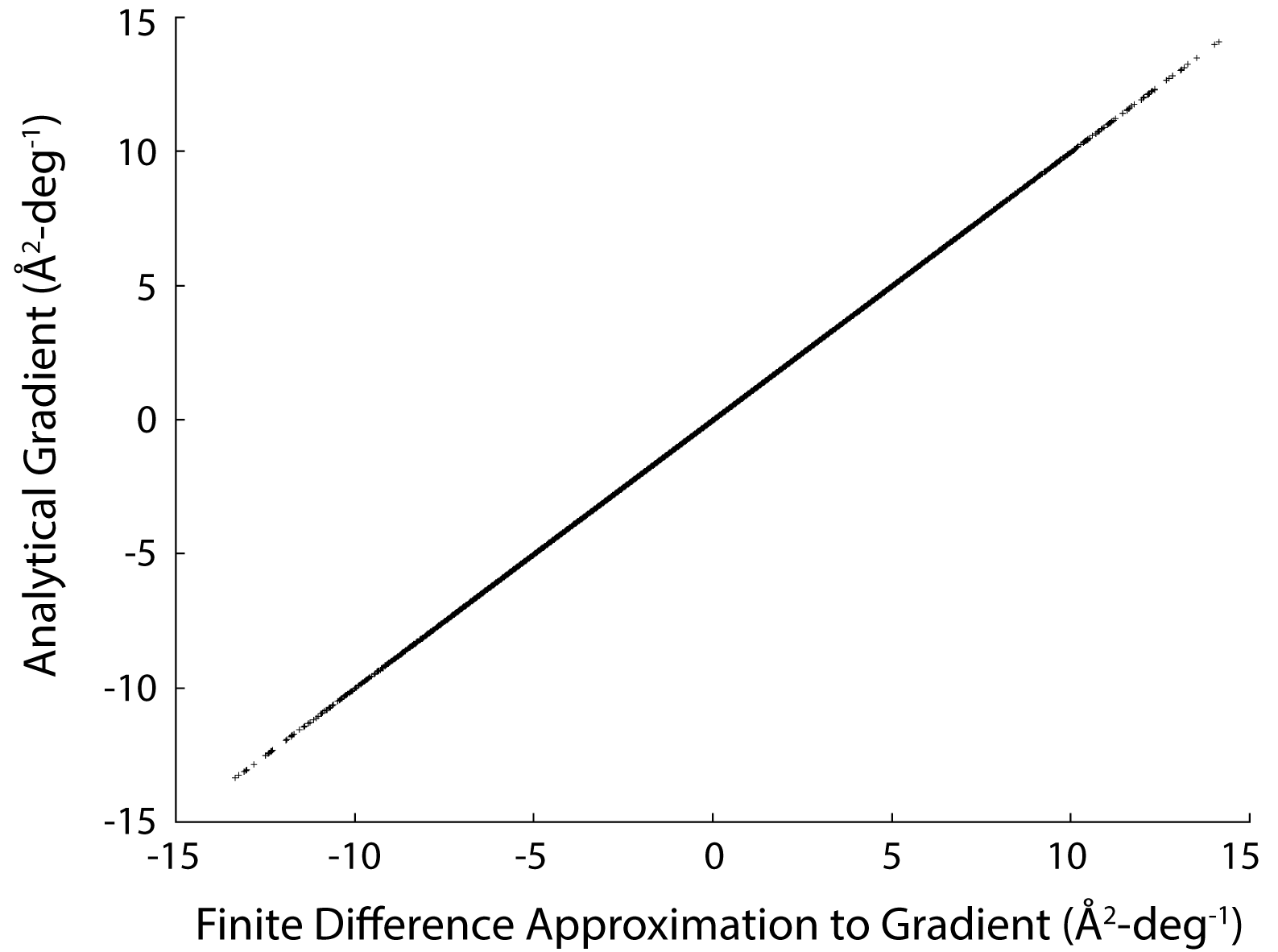


**Figure C. Comparison of contributions of single atom SASA changes to individual torsion gradients calculated analytically with values obtained by finite‑difference approximation.** Analytically calculated and numerically approximated values for the contributions of single atom SASA changes to the gradients at all torsional degrees of freedom were obtained for five structures (PDB codes: 1EAQA chain A, 1XODA chain A, 2Z5W chain A, 4K12 chain A, and 5COFA chain A). Over 1.1 million comparisons are included in the analysis. The average absolute difference between the analytical and approximate values is 2.8x10^-7^ Å^2^‑deg^‑1^.

**Figure D**


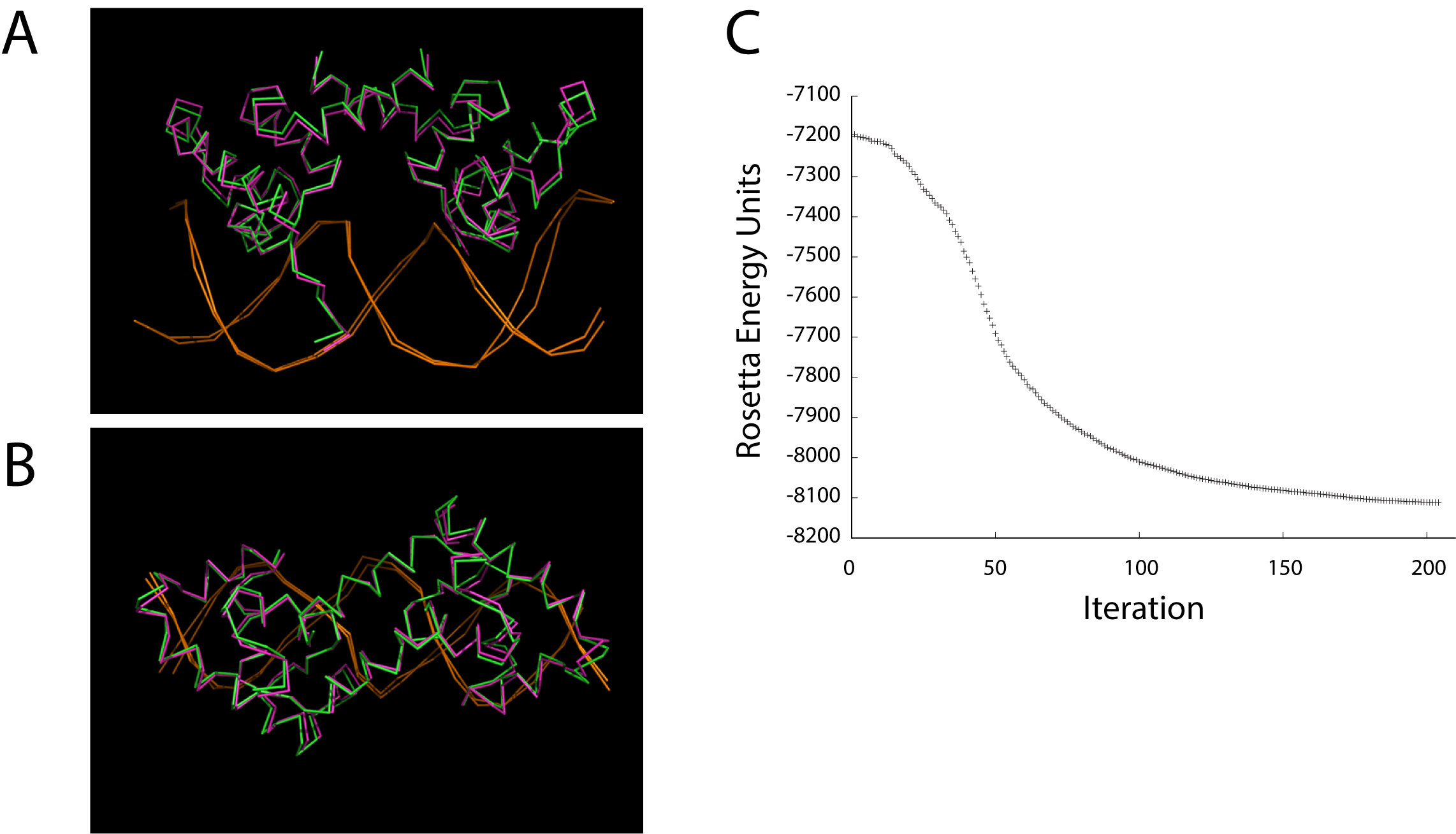


**Figure D. Minimization of lambda repressor-DNA complex.** An experimentally determined structure for the lambda repressor in complex with operator DNA (PDB code: 1LMB [1]) was subjected to energy minimization. The potential used consisted of the standard Rosetta force field [6] with the distance-dependent dielectic electrostatic, geometry‑dependent hydrogen bonding, and Lazaridis‑Karplus solvation terms [3] replaced with a polarizable multipole electrostatics potential (including a generalized Kirkwood treatment of solvent electrostatics [4]) and a surface area‑scaled cavity term. **A.** Side view of overlay of initial (green) and final (magenta) backbone traces resulting from minimization. **B.** Top view of backbone traces, using same color scheme as in panel A. **C.** Trajectory of energy of the protein‑DNA complex as a function of iteration during the Quasi‑Newton minimization algorithm.

**Supplemental Bibliography**

1. Beamer LJ, Pabo CO (1992) Refined 1.8 A crystal structure of the lambda repressor-operator complex. J Mol Biol 227: 177-196.

2. Nocedal J, Wright SJ (2006) Numerical optimization. New York: Springer. xxii, 664 p.

3. Lazaridis T, Karplus M (1999) Effective energy function for proteins in solution. Proteins 35: 133-152.

4. Schnieders MJ, Ponder JW (2007) Polarizable atomic multipole solutes in a generalized Kirkwood continuum. J Chem Theory Comput 3: 2083-2097.

5. Ponder JW, Wu C, Ren P, Pande VS, Chodera JD, Schnieders MJ et al. (2010) Current status of the AMOEBA polarizable force field. J Phys Chem B 114: 2549-2564.

6. Alford RF, Leaver-Fay A, Jeliazkov JR, O’Meara MJ, DiMaio FP, Park H et al. (2017) The Rosetta All-Atom Energy Function for Macromolecular Modeling and Design. J Chem Theory Comput 13: 3031-3048.
